# Oral cavity swabbing for diagnosis of group a Streptococcus: a prospective study

**DOI:** 10.1186/s12875-020-01129-6

**Published:** 2020-03-26

**Authors:** Limor Adler, Miriam Parizade, Gideon Koren, Ilan Yehoshua

**Affiliations:** 1 Tel Aviv University, Tel Aviv - Yafo, Israel; 2 Maccabi Healthcare Services, 27 Hamered St., Tel Aviv - Yafo, Israel; 3 Ariel University, Ariel, Israel; 4 Ben Gurion University, Beer Sheva, Israel

**Keywords:** Pharyngitis, Streptococcal infection, Diagnosis, Swabbing, Oral cavity, Family practice

## Abstract

**Background:**

Throat pain is a common complaint in the ambulatory setting. Diagnosis of group A Streptococcus is made with a culture, molecular test or a rapid antigen detection test from the tonsils or the posterior pharyngeal wall, while other areas of the oral cavity are considered unacceptable. The purpose of the study is to compare cultures from the tonsils or posterior pharyngeal wall (throat) with cultures from the oral cavity (mouth).

**Methods:**

A prospective study conducted in ambulatory care. Eleven family physicians collected 2 swabs (throat and mouth) from 200 consecutive patients who complaint about throat pain. Inclusion criteria were throat pain and Centor Criteria > 2. Exclusion criteria were tonsillectomy and age (< 3 or > 65 years old). Participants were later divided into two groups – pediatrics (3–18 years old) and adults (19–65 year old). Sensitivity and specificity of mouth culture were calculated, with throat culture considered the reference gold standard.

**Results:**

Between November 2017 and March 2019, 200 swabs were collected (101 adults and 99 children). In the adult group sensitivity of mouth culture was 72.1% (95% Confidence Interval [CI] 59.9–82.3%) and specificity was 100% (95% CI 92.7–89.4%-100%). In the pediatric group sensitivity of mouth culture was 78.3% (95% CI 65.8–87.9%) and specificity was 100% (95% CI 92.5–100%).

**Conclusion:**

Our study demonstrated higher sensitivity of mouth culture for GAS than previously published. This finding suggests that areas of the oral cavity that were considered as unacceptable sites for culture of GAS pharyngitis may be considered as acceptable swabbing sites.

**Trial registration:**

Trial registration: ClinicalTrials.gov, ID NCT03137823. Registered 3 May 2017.

## Background

Sore throat is a common complaint in the ambulatory setting. Most often, throat pain is a symptom of pharyngitis. Pharyngitis is caused by various etiologies, including viruses, bacteria and candida. The most important pathogen to recognize and diagnose when treating pharyngitis is group A Streptococcus (GAS) for the prevention of acute rheumatic fever and suppurative complications, to improve clinical symptoms and signs; for the rapid decrease in contagiousness; and for the reduction in transmission of GAS to close contacts of the patient [1, 2].

Clinical diagnosis alone is considered good but not enough due to broad overlap between the signs and symptoms of streptococcal and nonstreptococcal (usually viral) pharyngitis [3–6]. Therefore, except when obvious viral clinical features are present, a laboratory test should be performed in order to identify GAS as the pathogen [4]. Clinical scoring systems may help physicians decide which patients will benefit from a laboratory diagnostic test. The Centor Criteria includes four signs and symptoms; exudate or swelling of the tonsils, tender or swollen anterior cervical lymph nodes, temperature > 38 °C and the absence of cough [7]. Age was later added in the modified McIsaac score [3].

Definite diagnosis is made by laboratory tests, such as culture of the tonsils, which is considered the gold standard test, molecular test or rapid antigen detection test (RADT) with a sensitivity of 85–86% and a specificity of 95–96% in children [8, 9]. There are substantial differences among guidelines from different countries, regarding the need of culture or RADT for the diagnosis of GAS and regarding the need to prescribe antibiotic as a treatment. Guidelines from North America, France and Finland consider diagnosis of GAS necessary and treatment is advised. On the other hand, guidelines from Belgium, the Netherlands, England and Scotland consider throat pain as a self-limiting disease therefore culture and antibiotic treatment are not recommended. Israeli guidelines are in line with North American guidelines [10]. Accurate diagnosis is significant for two reasons; it is important to recognize patients with GAS for the prevention of acute rheumatic fever and suppurative complication. However, it is also essential to recognize patients without GAS for reducing unnecessary antibiotic prescription, which is a rising problem worldwide [11, 12].

### Site for optimal culture

The reliability of throat culture depends on several variables including the swabbing site within the pharyngeal-oral cavity, the use of anaerobic incubation conditions, selective culture plates and duration of incubation [1]. The Infectious Diseases Society of America (IDSA) states that throat swab specimens should be obtained from the surface of either tonsils (or tonsillar fossae) or the posterior pharyngeal wall. Other areas of the oral pharynx and mouth are not considered as acceptable sites [1].

Use of anaerobic incubation and selective culture media can increase the likelihood of detecting GAS if present [13]. The duration of incubation is also important, and the culture should be incubated at 35 °C for at least18–24 h prior to reading. When cultures are not held in complete anaerobic conditions, negative cultures should be reexamined after another 24 h to identify additional positive throat culture results [1, 14].

## Methods

The aim of this study was to compare cultures from swabs obtained from the buccal surface and the tongue (mouth culture) with cultures from swabs obtained from the tonsils and posterior oropharynx (throat culture) for the diagnosis of GAS pharyngitis in both children and adults.

We conducted a prospective study that compared mouth and throat cultures. The study was conducted at Maccabi Healthcare Services (MHS), the second largest healthcare fund in Israel. Eleven family physicians from 11 different MHS clinics in the southern district of Israel collected cultures from 200 consecutive patients, with a clinical picture of GAS pharyngitis who agreed to participate and signed informed consent.

### Study population

Inclusion criteria were a complaint of throat pain and a clinical picture of GAS pharyngitis (Centor Criteria > 2). Exclusion criteria were tonsillectomy and ages less than 3 years or over 65 years old. Patients suspected to be GAS carriers (i.e. had no symptoms) were not included in our study. Study population was divided into 2 age related groups including children (3–18 years old) and adults (19–65 years old).

### Study protocol

Participation in the study was offered to all suitable patients or their guardians, who visited the participating doctor’s clinics. Informed consent was signed by the patient or his guardian (for children) prior to sample collection. Two swabs were obtained from each patient; one from both sides of buccal surface and the front of the tongue (mouth culture) and the second from the tonsils and oropharynx (throat culture, the gold standard). RADT was not performed. No other additional tests were performed (including respiratory virus tests). All cultures were collected by physicians participating in the research.

Physicians treated patients according to clinical diagnosis and once received the results of the throat cultures, treatment was adjusted accordingly. Results of the mouth culture were documented in a separate file and were known solely to the laboratory manager and the primary researcher.

### Microbiological technique

All swabs were sent to MHS’ central laboratory with Amies agar transport media to optimize detection of bacteria. Transport time from the physician office to the laboratory was between 24 to 72 h. Swabs were cultured on Strep A selective agar (Novamed ltd. Israel). This substrate composed of 5% defibrinated sheep blood agar (DSBA) and additional antibiotics to prevent growth of normal bacterial flora of the upper respiratory tract (oxolinic, sulfamethaxazole plus trimethoprim acid, polymyxin B). In addition, an antibiotic disc (Bacitracin 0.2 IU) was added for aiding in the identification of GAS. Culture media were incubated at 35 °C under anaerobic conditions for 18–24 h. Identification of GAS was made by growth of typical β-haemolytic colonies, inhibited by the Bacitracin disc. Lancefield group A antigen test was not used. When difficulties recognizing GAS arose, a rapid test for the presence of specific Strep A antigen in the suspected colonies (StrepAstick, Novamed ltd. Israel) was used.

Molecular testing for detection of GAS is still not used in Israel, due to high costs.

### Statistical analysis

Sample size calculation was based on the assumption of 50% prevalence of GAS pharyngitis. Thus, a minimum sample size of 98 subjects (including 49 subjects having the disease) was required to achieve a minimum power of 80% (actual power = 81.0%) for detecting a change in the percentage value of sensitivity of a screening test from 0.50 to 0.70, based on a target significance level of 0.05 (actual *p* = 0.044) [15]. In this study we analyzed two different groups – children and adults. Thus sample size calculation applies to each group separately.

We used Stata, version 15.1 IC (StataCorp LP, College Station, Texas) to calculate exact binomial confidence intervals for sensitivity, specificity, positive predictive value (PPV) and negative predictive value (NPV). Likelihood ratios were calculated using the substitution formula, where 0.5 is added to all cell frequencies before calculation when there is a zero in one or more cells.

## Results

Eleven family physicians from MHS in the southern district of Israel collected swabs from 204 patients who agreed to sign informed consent, between November 2017 and March 2019. Four were excluded due to age (exclusion criteria were above 65 years or below 3 years old). Patients were divided to two groups: pediatric patients and adult patients.

### Pediatric patients group

In the pediatric group there were 99 patients, mean age of 9 years old (range: 3–18), 56:43 female/male ratio. Prevalence of GAS pharyngitis was 60.6% (95% CI: 50.3–70.3%). The results of throat and mouth cultures of pediatric patients are presented in Table 1. Sensitivity of the mouth culture was 78.3% (95% CI: 65.8–87.9%) with a specificity of 100% (95% CI: 92.5–100%). The PPV was 100% (95% CI: 92.7–100%) and the NPV was 75% (95% CI: 61.1–86%).

**Table 1:** Results of the throat and mouth cultures for group A streptococcus of pediatric and adults patients

		Throat culture			
		Positive		Negative	
		children	adults	children	adults
**Mouth culture**	Positive	47	49	0	0
	Negative	13	19	39	33

### Adult patients group

In the adult group there were 101 patients, mean age of 33 years old (range: 19 to 63), 73:28 female/male ratio. Prevalence of GAS pharyngitis was 63.3% (95% confidence interval [CI]: 57.3–76.3%). The results of throat and mouth cultures of adult patients are presented in Table 1. Sensitivity of the mouth culture was 72.1% (95% CI: 59.9–82.3%) with a specificity of 100% (95% CI: 89.4–100%). The positive predictive value (PPV) was 100% (95% CI: 92.7–100%) and the negative predictive value (NPV) was 63.5% (95% CI: 49–76.4%).

All patients in our study improved with antibiotic treatment provided in the community, and no patient was admitted to the hospital due to complications.

## Discussion

In our study we demonstrated mouth swab culture sensitivity of 78.3% for children and 72.1% for adults with a specificity of 100% in both groups. This finding supports the IDSA recommendation that the optimal site for culture is the posterior oropharynx or the tonsils. However, our findings challenge the statement that other sites in the oral cavity are not acceptable. The sensitivity of mouth culture in children was close to the sensitivity of RADT. For adults the sensitivity was slightly, but not significantly lower, possibly due to lower bacterial load in the oral cavity.

Swabbing the tonsils is a very common exam in the office of the primary care physician, with an unpleasant effect on children, causing distress and often gag reflex. Therefore, swabbing of the mouth may be a good alternative for the gold standard swabbing technique. With excellent specificity, if the result is positive, the physician can be sure he received the correct result. However, in case of a negative result, throat culture will be necessary to exclude the diagnosis, similar to common practice with RADT. This approach may be unacceptable for some physicians due to the need of a second visit. Further research is needed to test oral swabbing using RADT or molecular test with immediate results. In this approach, a positive result will be accepted and a negative result will require an oropharyngeal culture at the same visit.

### Strengths and limitations

Our study has several strengths, including large sample size of children and adults, the participation of 11 family physicians from different clinics, a single microbiological laboratory that examined all cultures and the use of newer microbiological techniques than those used in prior studies. A potential limitation of our study is the lack of RADT and molecular test in comparison to culture and lack of calculation of inter-clinician variation in swabbing accuracy.

### Comparison with existing literature

The IDSA recommendation about optimal site of throat culture is based on very limited amount of studies. Two studies conducted by Brien et al. and Gunn et al. in 1985 which examined a total number of 32 children [16, 17]. Both studies assessed children who were positive for GAS by throat culture and re-tested 1–4 days later in multiple sites of the oral cavity (see Table 2). Both studies showed significant superiority of cultures from optimal sites. However, mouth cultures yielded positive results in 42–63%.

**Table 2:** Studies conducted comparing mouth and throat cultures

Research and year of publication	Population studied	Sample size	Site of detection	Method of detection	Sensitivity	Reference standard
Brien et al. 1985	Children	12	9 different areas in the oral cavity	Cultures were inoculated on DSBA. Plates were incubated for 18–24 h at 37 °C in 10% CO2.	No sensitivity, specificity calculations. 63% of cultures from unacceptable sites showed some growth.	Tonsils and posterior pharyngeal wall
Gunn et al. 1985	children	20	7 different areas in the oral cavity	Cultures were inoculated on DBSA and on DBSA-SXT. Plates were incubated for 18–24 h at 35 °C in 5% CO2 in air.	No sensitivity, specificity calculations. Recovery of GAS from optimal vs. unsatisfactory sites were 53% vs. 24 and 75% vs. 42% on DBSA and DBSA-SXT respectively.	Tonsils, posterior pharynx and posterior tongue.
Fox et al. 2006	children	53	2 swabs (double swab collection) **throat swab (posterior pharynx and tonsils) **mouth swab (tongue and buccal mucosa)	**RADT (Abbott Signify Rapid Strep A test) **a DNA probe (a nucleic acid probe test) after 24 h **inoculation on DSBA-SXT (Becton Dickinson) in 5% CO_2_ at 35 °C for 48 h.	RADT – 19.4% (7.5–37.5%) DNA probe – 41.9% (23.9–60.9%) Culture (48 h) – 80.6% (62.5–92.5%)	positive culture or DNA probe of posterior pharynx/tonsils
Kelly L 2007	Children and adults	64	2 swabs **throat swab (posterior pharynx and tonsils) **buccal mucosa	Both swabs were tested using RADT (The SureStep Strep A (II) Test by Applied Biotech)	Sensitivity of mouth culture was 5.6%.	RADT from optimal sites.

*DSBA* 5% defibrinated sheep blood agar, *DSBA-SXT* 5% defibrinated sheep blood agar supplemented with sulfamethoxazole and trimetophrim, *RADT* rapid antigen detection test

As noticed in both studies, swabs from the oral cavity were not always negative and had some detection of GAS, though with unsatisfactory sensitivity. The most predominant limitations in both studies are the small numbers and the time interval between the first and second culture. In this time interval the bacterial load might have decreased causing a lower sensitivity for the “unsatisfactory” sites. Another limitation of both studies is the implication of results for today’s practice. Microbiological technology for cultures has improved and results from studies using older techniques are less relevant today.

Two later studies carried out in 2006–2007 further examined the question of optimal swabbing location (see Table 2). Fox et al. examined 53 children complaining of throat pain [18]. Each child underwent double swab collection, a throat swab (from the posterior pharynx and tonsils) and a mouth swab (the tongue and buccal mucosa). Each swab was tested by RADT, DNA probe and sent to the laboratory for culture. The sensitivities of rapid strep test, DNA probe and culture from the mouth (gold standard reference was positive culture or DNA probe of posterior pharynx/tonsils) were 19.4, 41.9 and 80.6%, respectively. The conclusion from this study was that despite IDSA recommendation, there may be some utility in special circumstance, such as a child who technically resists the deeper culture, to perform direct antigen tests or enhanced cultures on swab specimen taken from nonpharyngeal/nontonsillar sites.

Kelly examined 64 pediatric and adult patients [19]. Each patient was sampled from the pharynx and the buccal mucosa using 2 different swabs, both tested for GAS by RADT. The prevalence of RADT throat swabs positive for GAS was 12.5%. No buccal swabs were positive. The conclusion of this research was that swabbing the buccal mucosa using RADT was ineffective.

## Conclusion

Our study demonstrated higher sensitivity of mouth culture for GAS than previously published. This finding suggests that areas of the oral cavity that were considered as unacceptable sites for culture of GAS pharyngitis may be considered as acceptable swabbing sites. Culture from the oral cavity may be considered a first step in the diagnosis of GAS, though if negative, a definite diagnosis using throat culture still needs to be made. Further research is needed to check the sensitivity or RADT or molecular test using oral swab and in order to strengthen our results.

## Data Availability

The datasets used and/or analyzed during the current study are available from the corresponding author on reasonable request.

## Abbreviation

CI: Confidence interval
DSBA: Defibrinated sheep blood agar
GAS: Group A Streptococcus
IDSA: Infectious Diseases Society of America
MHS: Maccabi Healthcare Services
NPV: Negative predictive value
PPV: Positive predictive value
RADT: Rapid antigen detection test
